# Overt diabetes mellitus among newly diagnosed Ugandan tuberculosis patients: a cross sectional study

**DOI:** 10.1186/1471-2334-13-122

**Published:** 2013-03-05

**Authors:** Davis Kibirige, Richard Ssekitoleko, Edrisa Mutebi, William Worodria

**Affiliations:** 1 Department of Medicine, St. Raphael of St. Francis hospital Nsambya, P.O.BOX 7146, Kampala, Uganda; 2 Department of Medicine, Makerere University College of Health Sciences, Kampala, Uganda; 3 Infectious diseases unit, Mulago National referral and Teaching Hospital, Kampala, Uganda; 4 Endocrine unit, Mulago National referral and Teaching Hospital, Kampala, Uganda; 5 Pulmonology unit, Mulago National referral and Teaching Hospital, Kampala, Uganda

## Abstract

**Background:**

There is a documented increase of diabetes mellitus in Sub Saharan Africa, a region where tuberculosis is highly endemic. Currently, diabetes mellitus is one of the recognised risk factors of tuberculosis. No study has reported the magnitude of diabetes mellitus among tuberculosis patients in Uganda, one of the countries with a high burden of tuberculosis.

**Methods:**

This was a cross-sectional study conducted among 260 consenting adult patients with a confirmed diagnosis of tuberculosis admitted on the pulmonology wards of Mulago national referral and teaching hospital in Kampala, Uganda to determine the prevalence of diabetes mellitus and associated clinical factors. Laboratory findings as well as the socio-demographic and clinical data collected using a validated questionnaire was obtained. Point of care random blood sugar (RBS) testing was performed on all the patients prior to initiation of anti tuberculosis treatment. Diabetes mellitus was diagnosed if the RBS level was ≥ 200mg/dl in the presence of the classical symptoms of diabetes mellitus.

**Results:**

The prevalence of diabetes mellitus among the admitted patients with tuberculosis was 8.5%. Only 5 (1.9%) patients with TB had a known diagnosis of diabetes mellitus at enrolment. Majority of the study participants with TB-DM co-infection had type 2 diabetes mellitus (n=20, 90.9%).

At bivariate analysis, raised mean ALT concentrations of ≥80 U/L were associated with DM (OR-6.1, 95% CI 1.4-26.36, p=0.032) and paradoxically, HIV co-infection was protective of DM (OR-0.32, 95% CI 0.13-0.79, P=0.016). The relationship between DM and HIV as well as that with ALT remained statistically significant at multivariate analysis (HIV: OR- 0.17 95%CI 0.06-0.51, p=0.002 and ALT: OR-11.42 95%CI 2.15-60.59, p=0.004).

**Conclusion:**

This study demonstrates that diabetes mellitus is common among hospitalized tuberculosis patients in Uganda. The significant clinical predictors associated with diabetes mellitus among tuberculosis patients were HIV co-infection and raised mean serum alanine transaminase concentrations.

## Background

In 2011, the International Diabetes Federation (IDF) estimated that about 366 million people worldwide have diabetes mellitus (DM). 80% of these people live in the low and middle income countries where tuberculosis (TB) is highly prevalent [1]. According to the World Health Organisation (WHO), there were an estimated 8.8 million incident cases of TB globally in 2010 [2].

Currently, both TB and DM are of great public health importance globally especially in Sub Saharan Africa (SSA) due to the converging epidemics of both communicable and non communicable diseases. With a prevalence rate of TB of 193/100,000 in 2010, Uganda is one of the high burden TB countries in SSA [3]. There is also an observed trend of increasing prevalence of DM in Uganda. The estimated prevalence according to the International Diabetes Federation in 2010 was 2.2% [4].

Recent evidence advocates for bi-directional screening and care of TB and DM patients. This is because both morbidities adversely affect each other [5] and currently, there is plausible evidence from different studies to support the strong association between DM and TB [6,7]. Diabetic patients have impaired cell mediated immunity, renal failure, micronutrient deficiency and pulmonary microangiopathy, all of which increase their propensity to develop TB [8]. DM is also known to alter the clinical presentation of TB and its outcomes in terms of delayed sputum/culture conversion, case fatality and treatment failure [9].

TB co-infection is associated with poor glycemic control among DM patients. Reactionary hyperglycemia often accompanies chronic infections like TB due to the increased pro-inflammatory state and release of counter-regulatory stress hormones like epinephrine, cortisol and glucagon that are antagonistic of insulin [10]. Rifampicin, a very potent anti TB drug has also been shown to induce a transient early phase hyperglycemia owing to augmentation of intestinal glucose absorption [11].

This study sought to determine the prevalence of DM and the associated clinical factors among the adult TB patients admitted on the pulmonology wards of Mulago national referral and teaching hospital, Uganda.

## Methods

### Study site description

Mulago national referral and teaching hospital is located in Kampala, the capital city of Uganda and serves a population of about 2 million people. It is a 1,500 bed facility serving as a national referral hospital and teaching hospital for Makerere University College of Health Sciences, Uganda. The hospital has two adult pulmonology wards primarily for admission of patients with the varied pulmonary medical conditions like TB.

### Study methods

This was a cross sectional study in which adult patients with a confirmed diagnosis of TB admitted on the pulmonology wards of Mulago hospital were consecutively recruited during weekdays of the study period of September 2011 up to February 2012.

Patients enrolled into the study were ≥18 years of age, admitted on the pulmonology wards during the study periods and had a confirmed diagnosis of TB. All patients who were on anti TB drugs and those who could not offer informed consent were excluded from the study.

A confirmed diagnosis of TB was made if the patient presented with clinical symptoms suggestive of TB and at least one of the following: a positive sputum smear on Ziehl Nielsen or flourochrome (Auramine-O) stain for acid fast bacilli (AFB), a positive Xpert/RIF-TB test result, a positive sputum culture for TB and a histological diagnosis of TB on lymph node or pleural biopsy.

### Data collection

All patients gave informed consent prior to enrolment into the study. Information on the socio-demographic characteristics, medical history and laboratory variables of the consented eligible study participants was collected using pre-coded questionnaire forms. All patients underwent anthropometric measurements for estimation of the body mass index (BMI).

A capillary blood sample was obtained for measurement of the random blood sugar (RBS) level using a One Touch Ultra® glucometer from Johnson and Johnson Company, United Kingdom. Blood was also drawn for measurement of the serum albumin, renal function tests (serum urea and creatinine) for calculation of the glomerular filtration rate (GFR), liver function tests (alanine transaminase (ALT) and alkaline phosphatase (ALP) levels), HIV serology and CD4 counts. The normal values for the above tests were: serum albumin: 35–50 g/L, GFR: 90–120 ml/min/1.73 m [2], ALT: 0-80U/L and ALP: 30–129 U/L. All the above tests were done prior to initiation of anti TB drugs. The cockroft Gault formula below was used to estimate the glomerular filtration rate (GFR). Normal GFR was defined as an estimated GFR of ≥ 90 ml/min/1.73 m [2].

GFR = [(140-Age in years) × body weight in kg/ serum creatinine in mg/dl × 72] × 0.85 if female.

The diagnosis of DM was made basing on the recent American Diabetes Association (ADA) guidelines of a random or casual blood sugar level ≥ 200mg/dl in the presence of the classical symptoms of DM [12]. The classical symptoms of DM include: polyuria, polydipsia, polyphagia, generalised body weakness and progressive weight loss. Type 2 diabetic patients were defined as patients aged ≥30 years on oral hypoglycaemic drugs and/or insulin therapy while type 1 diabetic patients were those aged < 30 years on insulin monotherapy.

### Statistical analysis

Data was entered into EPI-INFO Version 6 and analysed using SPSS version 14. Patient’s characteristics were summarised with proportions and the continuous variables expressed in mean and standard deviation for normally distributed variables.

To determine associations between the different factors and RBS levels, the outcome variable, bivariate analyses using chi-square test was performed. For variables in which the cell values were less than 5, the Fischer’s exact test was used instead. Continuous variables were categorised into 2 groups based on the normal cut off values and then compared. Age was categorised into 2 groups using the cut of value of the average age.

Binary logistic regression analysis was performed to determine which factors were independently associated with DM among the study participants. Multivariate analysis was then performed and variables considered were those which had a conservatively set cut off p-value of <0.3 on bivariate analysis. A p-value of <0.05 and confidence intervals not including 1 were considered to be statistically significant.

Using the prevalence of 13.2% of DM among TB patients in Indonesia [13], with 80% power and a two-sided α < 0.05, a sample size of 176 study subjects was obtained. However, we enrolled 260 patients for the study.

### Ethical consideration

This study was approved by the department of Internal Medicine, Makerere College of Health Sciences and the Makerere University School of Medicine research and ethics committee, Uganda.

## Results

### Socio-demographic, clinical and laboratory characteristics of the patients

Of the 260 study participants enrolled, majority were male (n=146, 56.2%). The mean age of the study participants was 34.5 years (S.D 9.5). The youngest participant was 18 years and the eldest was 65 years. HIV co-infection was documented in 280 (80%) participants with 78% of them having CD4 counts < 200 cells/mm [3]. The mean BMI was 17.4 kg/m [2]. Sixty three (23.2%) and 197 (75.8%) participants had extra pulmonary TB (EPTB) and pulmonary TB (PTB) respectively. Fifty (19.2%) of the participants had TB relapse (Tables 1 and 2).

**Table 1:** Baseline socio-demographic factors and clinical characteristics of the study participants

**Characteristic**		**Frequency (n=260)**	**Percentage (%)**
Age (years) Mean (*SD) 34.7(9.5)			
	18-35	154	59.2
	36-50	91	35
	>50	15	5.8
**Gender**	Male	146	56.2
	Female	114	43.8
**Place of residence**	Rural	77	29.6
	Urban	183	70.4
**Level of education**	No formal education	18	6.9
	Primary level	168	64.6
	Secondary level	54	20.8
	Tertiary level	20	7.7
**Occupation**	Unemployed	58	22.3
	Self employed	34	13.1
	Unskilled	147	56.5
	Skilled	21	8.1
**Smoking status**	Former smokers	66	25.4
	Non smokers	194	74.6
**HIV infection**	Positive	208	80
**On HAART**	Yes	61	29
**Known diabetes mellitus**	Yes	5	1.9
**History of TB treatment.**	No	207	79.6
**Karnofsky score (%) Mean (SD) 61.5 (14.3)**			
	<70%	132	50.8
**Category of TB**	PTB	197	75.8
**Body mass index, kg/m**^**2 **^**Mean (SD) 17.4 (2.5)**			
	<18.5	181	69.6

* SD- Standard deviation.

**Table 2:** Laboratory findings of the study participants

**Laboratory characteristic**		**Frequency (n=260)**	**Percentage (%)**
**Serum albumin (g/L)**	**NR in g/L (35–50)**		
Mean (SD) 25.3(7.3)			
	<35	234	90
**CD4 count (cells/μl)**			
Mean (SD) 126.8 (161.8)			
	<200	163	78.4
**ALT level (U/L)	**NR in U/L (0–80)**		
Mean (SD) 17.4 (25.7)			
	≥80	9	3.5
***ALP level (U/L)	**NR in U/L (30–129)**		
Mean (SD) 160.9 (140.3)			
	≥129	109	41.9
****GFR (ml/min/1.73m^**2**^**)**	**NR in ml/min/1.73m**^**2 **^**(90–120)**		
Mean (SD) 86.3(26.1)			
	<90	69	26.5

* SD- Standard deviation NR- Normal range **ALT- Alanine transaminase *** ALP- Alkaline phosphatase ****GFR-Glomerular filtration rate.

### Prevalence of DM and the clinical characteristics of the TB-DM co-infected participants

DM was diagnosed in 22 study participants giving a prevalence of 8.5%. Twenty participants had type 2 DM while 2 had type 1 DM. Of the 22 participants, 5 (22.7%) had a known diagnosis of DM and all had poor glycemic control (defined as RBS level > 180 mg/dl) at baseline despite being on glucose lowering therapy. The background prevalence of DM on the medical units in the hospital during the study period was 6.4%.

The most frequent symptoms among the study participants with DM-TB co-infection were polyuria (90.9%), polydipsia (77.3%) and progressive weight loss (68.2%). The socio-demographic characteristics, category and episode of TB of the study participants did not significantly differ between the diabetic and non diabetic TB patients (Table 3).

**Table 3:** Bivariate analysis for the characteristics of the study participants

**Random Blood Sugar (RBS) level in mg/dl**				
**Characteristic**	**≥200, (n=22) n (%)**	**<200, (n=238) n (%)**	**OR 95%CI**	**P value**
**Age in years**				
Age≥35	17(6.5)	160(61.5)	1.66(0.59-4.66)	0.33
Age<35	5(1.9)	78(0.3)		
**Gender**				
Male	9(40.9)	137(57.6)	0.51(0.21-1.24)	0.13
Female	13(59.1)	101(42.4		
**Place of residence**				
Urban	15(68.2)	168(70.6)	0.89(0.35-2.28)	0.813
Rural	7(31.8)	70(29.4)		
**Occupation**				
Employed	17(77.3)	185(77.7)	0.97(0.34-2.76)	0.961
Unemployed	5(22.7)	53(22.3)		
**Smoking status**				
Former smoker	3(13.6)	63(26.5)	2.28(0.65-7.97)	0.141
Non smoker	19(86.4	175(73.5)		
**HIV serology**				
**HIV positive**	**13(59.1)**	**195(81.5)**	**0.32(0.13-0.79)**	**0.016**
**HIV negative**	**9(40.9)**	**43(18.5)**		
**Category of TB**				
δPTB	19(86.4)	178(74.8)	0.46(0.13-1.64)	0.225
§EPTB	3(13.6)	60(25.2)		
**Episode of TB**				
Newly diagnosed	18 (81.8)	192 (80.7)	2.13(0.61-7.5)	0.3
Relapsed TB	4(18.2)	46(19.3)		
*BMI, kg/m2				
≥17.5	7(2.6)	103(39.6)	0.61(0.24-1.6)	0.3
<17.5	15(5.7)	135(51.9)		
**GFR,ml/min				
GFR<90	**5**	**64**	1.25(0.44-3.53)	0.67
GFR≥90	17	174		
***ALT in U/L				
ALT≥80	3	6	6.1(1.4-26.36)	0.032
ALT<80	19	232		
**CD4/mm**^**3**^				
CD4≥350			0.82(0.1-6.7)	0.85
CD4<350				
****ALP-U/L.				
ALP≥12	8	102	0.76(0.31-1.88)	0.56
ALP<129	14	136		

SD- Standard deviation *BMI-Body mass index ** GFR-Glomerular filtration rate ***ALT- Alanine transaminase **** ALP- Alkaline phosphatase δPTB-Pulmonary tuberculosis §EPTB-Extra pulmonary tuberculosis.

At bivariate analysis, HIV co-infection was protective of DM (OR-0.32, 95% CI 0.13-0.79, P=0.016) and raised mean ALT concentrations were associated with DM (OR-6.1, 95% CI 1.4-26.36, p=0.032). For every unit change in the ALT category there was a 1.8 unit increase in the RBS (95% CI 0.35-3.3, p=0.02).

The relationship between DM and HIV as well as that with ALT remained statistically significant on multivariate analysis (HIV: OR- 0.17 95%CI 0.06-0.51, p=0.002 and ALT: OR-11.42 95%CI 2.15-60.59, p=0.004). On multivariate analysis, for every unit change in the ALT category, there was a 2.4 unit change in the random blood sugar (95%CI=0.77-4.1, p=0.04). Other variables considered in the multivariate analysis included age, sex, episode of TB infection (relapse or new infection), BMI, category of TB and smoking, though were not statistically significant.

The diabetic TB-HIV co-infected patients had lower baseline mean CD4 counts compared to non diabetic TB-HIV co-infected patients, although this was not statistically significant (Table 4).

**Table 4:** Predictor variables considered for association with DM at multivariate analysis

**Characteristic**	**Crude OR**	**95% CI**	**P value**	**Adjusted OR**	**95% CI**	**P value**
**HIV sero-status**	**0.32**	**(0.13-0.79)**	**0.016**	**0.17**	**(0.06-0.51)**	**0.002**
**ALT in U/L**	**6.10**	**(1.4-26.36)**	**0.032**	**11.42**	**(2.15-60.59)**	**0.004**
Gender	0.51	(0.21-1.26)	0.14	0.52	(0.17-1.55)	0.242
Smoking	2.28	(0.65-7.97)	0.14	2.64	(0.57-12.2)	0.214
TB category	0.46	(0.13-1.64)	0.225	0.56	(0.16-1.94)	0.36
TB episode	2.13	(0.61-7.5)	0.31	0.73	(0.48-7.4)	0.36
BMI	0.61	(0.24-1.6)	0.3	0.59	(0.21-1.64)	0.314

## Discussion

This study demonstrates that DM is highly frequent among admitted TB patients in Mulago hospital, Uganda. To our knowledge, this is the first study to examine this association in Uganda, one of the high burden TB countries in SSA. The reported prevalence of DM among the TB patients of 8.5% is significantly higher than the estimated prevalence of DM among the general population in Uganda (2.2%) [4] and the back ground prevalence of DM on the medical units in the hospital during the study period (6.4%).

The documented prevalence of DM among TB patients in the African studies published between 1980 and 2006 varies between 2.1%-6.7% [14-17]. The observed prevalence of DM among TB patients in our study is comparable to that reported from Nigeria [16] and Tanzania [17] but higher than what was noted in South Africa [14] and Guinea Conakry [15].

This heterogeneity in the above results could probably be explained by the varied techniques used to diagnose DM among the TB patients and the probable effects of co morbidities like HIV. Largely, an oral glucose tolerance test (OGTT) was used in diagnosing DM in most African studies [14,16,17]. Balde et al. used a fasting capillary blood test to diagnose DM among TB patients in Guinea Conakry [15]. In our study, the diagnosis of DM was made basing on a random or casual capillary blood test. No particular method of diagnosing DM among TB patients has been advocated for. Either a random blood sugar (RBS), fasting blood sugar (FBS), OGTT or glycated haemoglobin (HbA1c) test can be used alone or in combination [5].

Similar studies from other parts of the world have reported remarkably higher prevalence of DM among TB patients. In Asia and the Middle East, the documented prevalence varies from 9.5%-44% [13,18-23] and 11.9%-27% [24-26] respectively. A multi centre study done in Texas, USA and Mexico reported prevalence of 39% and 36% respectively [27]. This higher prevalence could probably be due to the higher background prevalence of DM in the general population in those respective countries.

Type 2 DM was the most frequent type of DM encountered among the study participants with DM-TB co-infection (90.9%). A similar observation has been documented in most similar studies [13,15,18]. This could probably be due to the higher proportions of people with type 2 DM compared to type 1 DM in most general populations.

Five (1.9%) of the study participants at enrolment had a prior diagnosis of DM and on assessment to determine the extent of glycemic control, they all had poor glycemic control which we defined as a RBS level ≥ 180 mg/dl. TB infection is often associated with a transient stress induced hyperglycemia which results into suboptimal glycemic control among diabetic patients. It usually resolves following TB therapy [28].

This form of reactionary hyperglycemia can also lead to over diagnosis of DM among TB patients. Studies by Alisjahbana et al. [13], Oluboyo et al. [16] and Mugusi et al. [17] demonstrated improvement in the glycemic status of some patients following TB treatment. However in our study, we did not perform a repeat assessment of the glycemic status among the newly diagnosed DM patients during the course of TB therapy.

HIV co-infection and raised mean serum ALT concentrations were noted to be independently associated with DM among TB patients in our study. In contrast to available literature and findings from other studies [29-33], HIV infection appeared protective in our study. A probable explanation for this is that most of our HIV positive patients were taking cotrimoxazole prophylaxis, a drug which has been found to cause hypoglycaemic effects in some patients [34]. However, this relationship between HIV and DM in our study needs to be interpreted with caution. This is because a study done in this similar setting to determine glycemic levels in patients with severe sepsis (80% of whom also had HIV infection and were on cotrimoxazole prophylaxis) revealed no statistically significant association between HIV infection and hyperglycemia (OR-0.97, 95%CI 0.57-1.62) [35].

Raised mean serum ALT concentrations in TB/DM patients have not been described in any similar studies. However, since majority of the study participants had HIV co-infection, an elevated mean ALT concentration of >66 U/L prior to diagnosis of DM was noted to be significantly associated with DM among HIV infected patients in one case control study performed in an urban HIV clinic in the USA [33].

A raised mean serum ALT concentration is a strong predictor of insulin resistance [36]. It also principally reflects direct hepatocellular damage or liver dysfunction. Liver dysfunction secondary to underlying hepatitis C and hepatosteatosis have been demonstrated to be associated with DM [37-40].

Hepatitis C infection is associated with insulin resistance owing to an increased pro-inflammatory state and production of cytokines especially tumour necrosis factor α and interleukin 6. These inhibit transcription of the glucose transporter-4 and peroxisome proliferator –activated receptor γ. Insulin resistance results into alteration in lipid metabolism and deposition in the hepatocytes. Hepatosteatosis often develops later. Hepatitis C co-infection is also associated with autoimmune pancreatic beta cell damage leading to DM [37-40]. In addition to hepatitis C infection, human herpes virus type 8 (HHV-8), a highly prevalent virus among HIV infected patients that causes with kaposi sarcoma has also been demonstrated to be linked to ketosis prone type 2 DM, an atypical form of DM commonest among black Africans [41]. In our study however, we did not assess for presence of hepatosteatosis, hepatitis C or HHV-8 co-infection among the study participants.

Majority of the similar studies have reported increasing age, overweight or obesity [15,19,20,27] and male gender [20] as the clinical factors associated with DM among TB patients. Sedentary lifestyle and family history of DM and obesity were also noted to be independently associated with DM in the study done in Guinea Conakry [15]. These clinical factors were not significantly associated with DM among our study participants.

### Study limitations

We acknowledge important limitations in our study. Only one RBS estimation was used to diagnose DM hence leading to a possibility of overestimating of the prevalence of DM among our study participants since patients with reactive hyperglycemia could have been included. Use of an OGTT could have diagnosed more patients with borderline DM.

Although the study adjusted for some of the confounding factors, the role of residual confounding factors for the association between DM and TB cannot be ruled out. In addition, due to the cross sectional nature of the study, the temporality between the TB and DM could not be ascertained.

As similar findings have been documented among TB patients in other parts of the world, we believe that our findings are generalisable and hence we recommend that patients diagnosed with TB should routinely have their blood sugar levels assessed in order to enable timely diagnosis and optimal management of DM.

## Conclusion

DM is a frequent co morbid condition among TB patients in Uganda. HIV co-infection and raised mean serum ALT concentrations are independently associated with DM among TB patients. We recommend routine screening for DM among TB patients in Uganda especially those with raised mean serum ALT concentrations of ≥80 U/L. Further prospective studies to examine the effects of DM on the clinical outcomes among TB-DM co-infected patients in Uganda are warranted.

## Abbreviations

DK: Davis Kibirige; RS: Richard Ssekitoleko; EM: Edrisa Mutebi; WW: William Worodria

## Competing interests

The authors declare that they have no competing interests.

## Authors’ contributions

Concept development: DK, EM, RS and WW, Data collection: DK, Supervision of the study: EM and WW, Data interpretation and revision of the all the drafts: DK, EM, RS and WW. All authors read and approved the final manuscript.

## Pre-publication history

The pre-publication history for this paper can be accessed here:

http://www.biomedcentral.com/1471-2334/13/122/prepub

## References

[B1] International Diabetes Federation Diabetes Atlas http://wwwidforg/diabetesatlas/5e/ Accessed CIon 26/06/2012.

[B2] Global tuberculosis control: WHO report 2011

[B3] http://www whoint/tb/data Accessed on 6/6/2012

[B4] The International Diabetes Federation Atlas 2010 4

[B5] Ottmani S Murray M Jeon C Consultation meeting on tuberculosis and diabetes mellitus: meeting summary and recommendations Int J Tuberc Lung Dis 2010 14 12 1513 1517 21180207

[B6] Faurholt-Jepsen D Range N PrayGod G Diabetes Is a Risk Factor for Pulmonary Tuberculosis: A Case–control Study from Mwanza, Tanzania PLoS One 2011 6 8 e24215 21912626 10.1371/journal.pone.0024215 PMC3166171

[B7] Jeon C Murray M Diabetes Mellitus Increases the Risk of Active Tuberculosis: A Systematic Review of 13 Observational Studies PLoS Med 2008 5 7 1091 1101 10.1371/journal.pmed.0050152 PMC2459204 18630984

[B8] Harriesa A Billo N Kapurc A Links between diabetes mellitus and tuberculosis: should we integrate screening and care? Trans R Soc Trop Med Hyg 2009 103 1 2 18809194 10.1016/j.trstmh.2008.08.008

[B9] Stevenson C Forouhi N Roglic G Williams B Lauer J Dye C Diabetes and tuberculosis: the impact of the diabetes epidemic on tuberculosis incidence BMC Publ Health 2007 7 234 10.1186/1471-2458-7-234 PMC2001194 17822539

[B10] Van-Cromphaut S Vanhorebeek I Van-den-Berghe G Glucose metabolism and insulin resistance in sepsis Curr Pharm Des 2008 14 1887 1899 18691100 10.2174/138161208784980563

[B11] Takasu N Yamada T Miura H Rifampicin-induced early phase hyperglycemia in humans Am Rev Respir Dis 1982 125 23 27 7039435 10.1164/arrd.1982.125.1.23

[B12] American Diabetes Association Position statement- Standards of Medical Care in Diabetes Diabetes Care 2012 35 S11 S63 22187469 10.2337/dc12-s011 PMC3632172

[B13] Alisjahbana B Van-Crevel R Sahiratmadja E **Diabetes mellitus is strongly associated with tuberculosis in Indonesia** Int J Tuberc Lung Dis 2006 10 6 696 700 16776459

[B14] Marais R Diabetes Mellitus in Black and Coloured Tuberculosis patients S Afr Med J 1980 57 483 484 7368009

[B15] Baldé N Camara A Camara L Diallo M Kaké A Bah-Sow O Associated tuberculosis and diabetes in Conakry, Guinea: prevalence and clinical characteristics Int J Tuberc Lung Dis 2006 10 9 1036 1040 16964797

[B16] Oluboyo P Erasmus R The significance of glucose intolerance in pulmonary tuberculosis Tubercle 1990 71 135 138 2219464 10.1016/0041-3879(90)90010-6

[B17] Mugusi F Swai A Alberti K McLarty D Increased prevalence of diabetes mellitus in patients with pulmonary tuberculosis in Tanzania Tubercle 1990 71 271 276 2267680 10.1016/0041-3879(90)90040-f

[B18] Zhang Q Xiao H Sugawara I **Tuberculosis complicated by diabetes mellitus at Shangai Pulmonary hospital** China Jpn J Infect Dis 2009 62 390 391 19762992

[B19] Wang P Lin R Epidemiological features of diabetics among tuberculosis patients in Taiwan J Infect Dis Antimicrob Agents 2000 17 101 105

[B20] Suleiman S Aweis D Mohamed A RazakMuttalif A Moussa M Role of Diabetes in the Prognosis and Therapeutic Outcome of Tuberculosis Int J Endocrinol 2012 10 1 6 10.1155/2012/645362 PMC3337603 22570649

[B21] Li L Lin Y Mi F Screening of patients with tuberculosis for diabetes mellitus in China Trop Med Int Health 2012 17 10 1294 1301 10.1111/j.1365-3156.2012.03068.x 22830945

[B22] Viswanathan V Kumpatla S Aravindalochanan V Prevalence of Diabetes and Pre-Diabetes and Associated Risk Factors among Tuberculosis Patients in India PLoS One 2012 7 7 e41367 22848473 10.1371/journal.pone.0041367 PMC3406054

[B23] Balakrishnan S Vijayan S Nair S High Diabetes Prevalence among Tuberculosis Cases in Kerala, India PLoS One 2012 7 10 e46502 23077512 10.1371/journal.pone.0046502 PMC3471898

[B24] Golsha R Shiraz R Shafiee A Najafi L Dashti M Roshandel G Pulmonary Tuberculosis And Some Underlying Conditions In Golestan Province Of Iran, During 2001–2005 J Clin Diagn Res 2009 3 1302 1306

[B25] Singla R Khan N Al-Sharif N Al-Sayegh M Shaikh M Osman M Influence of diabetes on manifestations and treatment outcome of pulmonary TB patients Int J Tuberc Lung Dis 2006 10 1 74 79 16466041

[B26] Jabbar A Hussain S Khan A Clinical characteristics of pulmonary tuberculosis in adult Pakistani patients with co-existing diabetes mellitus East Mediterr Health J 2006 12 5 522 527 17333789

[B27] Restrepo B Camerlin A Rahbar M Cross-sectional assessment reveals high diabetes prevalence among newly-diagnosed tuberculosis cases Bull World Health Organ 2011 89 352 359 21556303 10.2471/BLT.10.085738 PMC3089389

[B28] Shamoon M Hendlei R Sherwin R Synergistic interaction among anti-insulin hormones in the pathogcncsis of stress hyperglyccmia in humans J Clin Endo Metab 1981 52 1235 1240 10.1210/jcem-52-6-1235 7014600

[B29] Kalra S Kalra B Agrawal N Unnikrishnan A Understanding diabetes in patients with HIV/AIDS Diabetol Metab Syndr 2011 3 2 21232158 10.1186/1758-5996-3-2 PMC3025836

[B30] Petoumenos K Worm S Fontas E Predicting the short-term risk of diabetes in HIV-positive patients: the Data Collection on Adverse Events of Anti-HIV Drugs (D:A:D) study J Int AIDS Soc 2012 15 17426 23078769 10.7448/IAS.15.2.17426 PMC3494158

[B31] El-Sadr W Mullin C Carr A Effects of HIV disease on lipid, glucose and insulin levels: Results from a large antiretroviral-naive cohort HIV Med 2005 6 114 121 15807717 10.1111/j.1468-1293.2005.00273.x

[B32] Kilby J Tabereaux P Severe hyperglycemia in an HIV clinic: Pre-existing versus drug-associated diabetes mellitus J Acquir Immune Defic Syndr Hum Retrovirol 1998 17 46 50 9436758 10.1097/00042560-199801010-00007

[B33] Yoon C Gulick R Hoover D Case–control study of diabetes mellitus in HIV-infected patients J Acquir Immune Defic Syndr 2004 37 1464 1469 15602124 10.1097/01.qai.0000137373.26438.18

[B34] Strevel E Kuper A Gold W Severe and protracted hypoglycaemia associated with co-trimoxazole use Lancet Infect Dis 2006 6 3 178 182 16500599 10.1016/S1473-3099(06)70414-5

[B35] Ssekitoleko R Jacob S Banura P Hypoglycemia at admission is associated with inhospital mortality in Ugandan patients with severe sepsis Crit Care Med 2011 39 2271 2276 21666451 10.1097/CCM.0b013e3182227bd2 PMC3730257

[B36] Chung R Casson D Murray G Alanine aminotransferase levels predict insulin resistance in HIV lipodystrophy J Acquir Immune Defic Syndr 2003 34 534 536 14657767 10.1097/00126334-200312150-00015

[B37] Mason A Lau J Hoang N Association of diabetes mellitus and chronic hepatitis C virus infection Hepatology 1999 29 328 333 9918906 10.1002/hep.510290235

[B38] Ali S Abera S Mihret A Abebe T Association of Hepatitis C Virus Infection with Type II Diabetes in Ethiopia: A Hospital-Based Case–control Study Interdiscip Perspect Infect Dis 2012 10 1155 10.1155/2012/354656 PMC3461610 23049551

[B39] Naing C Mak J Ahmed S Maung M Relationship between hepatitis C virus infection and type 2 diabetes mellitus: Meta-analysis World J Gastroenterol 2012 18 14 1642 1651 22529694 10.3748/wjg.v18.i14.1642 PMC3325531

[B40] Masini M Campani D Boggi U Hepatitis C Virus Infection and Human Pancreatic beta-Cell Dysfunction Diabetes Care 2005 28 4 940 941 15793203 10.2337/diacare.28.4.940

[B41] Sobngwi E Choukem S Agbalika F Ketosis-Prone Type 2 Diabetes Mellitus and Human Herpes virus 8 Infection in Sub-Saharan Africans JAMA 2008 299 23 2770 2776 18560004 10.1001/jama.299.23.2770

